# Toward a better understanding of microalgal photosynthesis in medium polluted with microplastics: a study of the radiative properties of microplastic particles

**DOI:** 10.3389/fbioe.2023.1193033

**Published:** 2023-05-04

**Authors:** Limin Yang, Chunyang Ma

**Affiliations:** ^1^ School of Life Sciences, Jiangsu University, Zhenjiang, Jiangsu, China; ^2^ School of Advanced Manufacturing, Nanchang University, Nanchang, China; ^3^ NUS Environmental Research Institute, National University of Singapore, Singapore, Singapore

**Keywords:** microalgal photosynthesis, microplastics, radiative properties, light transfer, scattering

## Abstract

Due to the wide presence of microplastics in water, the interaction between microplastic particles and microalgae cells in medium merits the attention of researchers. Microplastic particles can impact the original transmission of light radiation in water bodies since the refractive index of microplastics is different from that of water bodies. Accordingly, the accumulation of microplastics in water bodies will certainly impact microalgal photosynthesis. Therefore, experimental measurements and theoretical studies characterizing the radiative properties of the interaction between light and microplastic particles are highly significant. The extinction and absorption coefficient/cross-section of polyethylene terephthalate and polypropylene were experimentally measured using transmission and integrating methods in the spectral range of 200–1,100 nm. The absorption cross-section of PET shows remarkable absorption peaks in the vicinity of 326 nm, 700 nm, 711 nm, 767 nm, 823 nm, 913 nm, and 1,046 nm. The absorption cross-section of PP has distinctive absorption peaks near 334 nm, 703 nm, and 1,016 nm. The measured scattering albedo of the microplastic particles is above 0.7, indicating that both microplastics are scattering dominant media. Based on the results of this work, an in-depth understanding of the interaction between microalgal photosynthesis and microplastic particles in the medium will be obtained.

## 1 Introduction

To attenuate the greenhouse effect worldwide, microalgae, in recent years, are intensively employed to capture atmospheric CO_2_ or reduce CO_2_ emission in industries. In the practice, to improve the performance of microalgae in CO_2_ capture, photosynthesis should be enhanced. Therefore, considerable emphasis has been centered on the optimization of environmental parameters to create suitable conditions for microalgal photosynthesis. Previous studies have studied the effects of turbidity, temperature, pH, and hardness of medium on microalgal growth and metabolisms. However, few research works have focused on the interaction between microplastic particles in medium and microalgal photosynthesis.

Plastic products have penetrated all aspects of modern human life due to their excellent performance. Microplastics originate from anthropogenic production and disposal of waste plastic products, which have harmful potential impacts on human and ecosystem health (Leal Filho et al., 2019; Jiang et al., 2022). Currently, the impact of microplastics on human health and ecosystem balance is unclear and difficult to estimate since research on microplastics is still in its infancy (Leslie et al., 2022). Nevertheless, the presence of microplastic particles has been observed in natural waters, wastewater, and underground water.

The common plastic types include polyethylene terephthalate (PET), polypropylene (PP), polyethylene (PE), polystyrene (PS), polyvinyl chloride (PVC), polyethylene naphthalate (PEN), and polyamide-12 (PA12) (Hale et al., 2020). The global average percentage of plastics recycled is low, with the majority of plastics entering the natural environment through incineration, burial, or direct disposal (Hale et al., 2020). For example, more than half of the garbage that enters the ocean is plastic waste. Plastic waste breaks down in interaction with the marine environment resulting in microplastics (Kane et al., 2020). Microplastics are widely distributed in nature through oceanic circulation and wind transport (Campanale et al., 2020). Microplastics entering water bodies, both freshwater and marine, will have an impact on the original water body light radiative transfer to the extent that it affects the biological physiology in the water body (Issac and Kandasubramanian, 2021). The radiative properties of microplastics will have an important impact on the transfer of light radiation in the water body and further influence microalgal photosynthesis.

Moreover, microplastics are considered to be one of the most common microplastic contaminants (Manzoor et al., 2020; Kinigopoulou et al., 2022). However, recycling microplastic contaminant particles from the ocean and applying them to solar collectors not only helps to improve marine ecology but also improves the heat transfer performance of solar collectors. In addition, research on the radiative properties of microplastic particles can also be employed to enhance microalgal photosynthesis in a medium polluted with microplastics (Hamd et al., 2022). Research has shown that the addition of nano- and micron-particle suspensions to the conventional solar collector can improve its heat transfer performance (Devendiran and Amirtham, 2016; Turkyilmazoglu, 2016). Rubin (Rubin, 1982) studied the radiative properties of PET films in the infrared band based on optical constants. Ouchi et al. (1996) reported the absorption spectra of PET and PEN films in soft X-ray regions by using polarized synchrotron radiation. Liu’s group proposed an improved transmission method for measuring the extinction characteristics of particle suspensions (Li et al., 2015a; Li et al., 2016). The combined double optical pathlength transmission with the ellipsometry method was applied to obtain the optical constants of transparent materials (Li et al., 2015b; Li et al., 2017a). Kameya and Hanamura (Kameya and Hanamura, 2011) proposed a new transmittance measurement technique to measure the absorption characteristics of the nanoparticle suspension at Vis-NIR wavelengths. The absorption coefficient of the nanoparticle suspensions was obtained by spectral transmittance, and the Kramers-Kronig relation was used to acquire the refractive index of the nanofluid. Zhang et al. (2020a); Zhang et al. (2020b) experimentally measured the spectral optical constants of seven different polymers in the range of 0.4–20 μm. Currently, there is a lack of research on the radiative properties of PET and PP particles in the visible and near-infrared wavelength bands. The radiative properties of microplastic in the visible wavelength band have important applications for the investigation of light radiative transfer in aqueous ecosystems.

In this study, the spectral normal transmission and hemispherical transmittance measurements of the radiative properties of PET and PP microplastic suspensions were conducted. The extinction coefficients/cross-sections, absorption coefficients/cross-sections, and SPF of the two microplastic particles in the spectral range of 200–1,100 nm were presented. Furthermore, the characteristics of the radiative properties of microplastic particles were analyzed and summarized. It is expected that this work could provide important knowledge to improve microalgal photosynthesis in a medium polluted with microplastic particles.

## 2 Light transfer theory

When the light beam is directed into the microplastic suspension, the light will be absorbed and scattered. The interaction between light and microplastic particles is described by the radiative properties of particles. The radiative transfer process can be quantitatively governed by the light transfer equation (LTE), which can be generally written as (Modest and Mazumder, 2021),

$$s\cdot \nabla I_{\lambda }\left(r,s\right)=-\beta_{\lambda }I_{\lambda }\left(r,s\right)+\frac{\kappa_{s,\lambda }}{4\pi }\int_{4\pi }I_{\lambda }\left(r,s^{\prime }\right)\Phi \left(s^{\prime },s\right)d\Omega^{\prime }$$ (1)

with the emitting term neglected for the spectral region studied in this work. Here, $I_{\lambda }$ indicates the spectral radiation intensity, $\beta_{\lambda }=\kappa_{a,\lambda }+\kappa_{s,\lambda }$ indicates the spectral extinction coefficient, $\kappa_{a,\lambda }$ indicates the spectral absorption coefficient, $\kappa_{s,\lambda }$ indicates the spectral scattering coefficient, and $\Omega^{\prime }$ indicates the solid angle. The SPF $\Phi \left(s^{\prime },s\right)$ denotes the probability that the scattered radiation in the direction of $s^{\prime }$ enters the direction of s . The spectral asymmetry factor $g_{\lambda }$ is defined as the mean cosine of the SPF (Modest and Mazumder, 2021)

$$g_{\lambda }=\frac{1}{4\pi }\int_{4\pi }\Phi_{\lambda }\left(s^{\prime },s\right)\cos\theta d\Omega$$ (2)

where the θ is the angle between direction $s^{\prime }$ and s . The spectral absorption and extinction coefficients can be expressed separately using cross-sections as follows (Modest and Mazumder, 2021)

$$\kappa_{a,\lambda }=C_{abs,\lambda }N$$ (3)

$$\beta_{\lambda }=C_{ext,\lambda }N$$ (4)

where $C_{abs,\lambda }$ and $C_{ext,\lambda }$ indicate the spectral absorption and extinction cross-sections of microplastics, respectively. N indicates the particle number density of microplastic particles (m^-3^). It is clear from the LTE that the radiative properties of microplastic are the basic parameters for solving the light transfer process and distribution.

## 3 Experimental methods

### 3.1 Extinction and absorption coefficient measurements

The extinction coefficient of microplastic particles was obtained from the experimentally measured normal transmittance of the microplastic suspensions in cuvettes. The schematic of the microplastic sample for subsequent normal and hemispherical transmittance measurements is provided in the Supplementary Figure S1. The sample thickness is $L_{2}=10$ mm and the glass thickness is $L_{1}=L_{3}=1.5$ mm in the experiment. The light source was corrected to the collimated incident light by using a collimator. The experimental measurement procedure is as follows: The reference spectral light transmittance $T_{n,\lambda ,ref}$ was measured by adding deionized water to a quartz glass cuvette. Then, the experimental spectral light transmittance $T_{n,\lambda }$ was measured by adding the microplastic powder suspension to a quartz glass cuvette. The extinction coefficient of the microplastic suspension can be obtained from the normal-normal transmittance as follows (Pilon et al., 2011)

$$\beta_{\lambda }=-\frac{1}{L}\ln\frac{T_{n,\lambda }}{T_{n,\lambda ,ref}}$$ (5)

where *L* denotes the thickness of the quartz glass cuvette. Afterward, the extinction cross-section of the microplastic particles can be obtained by dividing their extinction coefficient by the particle number concentration. In the transmission method for measuring the extinction coefficient, it is important to note that the transmittance measurement of the reference sample can be used as a means to eliminate the experimental deviation caused by multiple reflections of the three-layer optical transmission system (Li et al., 2016).

The spectral normal hemispherical transmittance of the microplastic suspension was measured by using the integrating sphere technique. An integrating sphere with an 8 cm diameter was used to collect the scattered energy. The interior of the integrating sphere was coated with a highly reflective material, polytetrafluoroethylene, which has a good Lambertian reflective property and over 98% reflectivity, allowing efficient measurement of the transmitted energy in all directions. The formula of the absorption coefficient measurement can be generally written as (Pilon et al., 2011)

$$\kappa_{a,\lambda }=-\frac{1}{L}\ln\frac{T_{h,\lambda }}{T_{h,\lambda ,ref}}$$ (6)

where $T_{h,\lambda }$ is the reference spectral light transmittance, which was measured from a quartz glass cuvette filled with deionized water, and $T_{h,\lambda ,ref}$ represents the experimental spectral light transmittance measured from a cuvette with microplastic powder suspension. Strictly speaking, the above formulas for measuring extinction and absorption coefficients are only applicable to the condition of single scattering. The single scattering condition can only be satisfied approximately in practical experiments. It has been shown that the error introduced by using the above equations is within the experimentally allowed range when the optical thickness is less than one. It should be also noted that the experimental deviation owing to the effect of backscattered light can be neglected, which is due to the backward-scattering light being sufficiently smaller than the forward hemispherical transmitted light (Pilon et al., 2011; Ma et al., 2018a). The size parameter of the microplastic particle is large in the studied visible to near-infrared spectral region, so the forward scattering properties of the particles are significant, and the backward scattering can be neglected (Jonasz and Fournier, 2011). A deuterium-halogen two-in-one light source was used in the experiment, which could continuously output a stable spectrum ranging from 190 to 2,500 nm. A spectrometer of type MAX2000-Pro was used to measure the transmitted signal.

### 3.2 Sample preparation

A common approach called the two-step method can be used to configure microplastic particle suspensions. Among the various methods of nanofluid preparation, the two-step method serves as one of the most widely used pathways for the preparation of particle suspensions (Yu and Xie, 2012). Firstly, the micro and nanopowder particles are prepared using physical or chemical methods, and secondly, the nanopowders are dispersed uniformly to form a stable particle suspension using external stirring. In this study, the PET and PP microplastic powder was directly purchased from the Hua Chuang Plastic Chemical Co. Microplastics are not miscible with water, so the approach described in existing literature was employed to disperse the microplastic particles uniformly in deionized water (Kumar and Arasu, 2018; Li et al., 2020). We weighed a certain mass of microplastic powder and mixed it with deionized water. We added a small amount of dispersant (Tarleton X-100 emulsifier OP) and then stirred with a magnetic stirrer for half an hour to form a microplastic suspension. Additionally, the microplastic suspension was subjected to ultrasonic shaking for more than half an hour with an ultrasonic cleaner to achieve dispersion and stabilize the microplastic suspension. The effect of dispersant on the measurement of the radiative properties of microplastic particles is negligible because the dose of dispersant is very small and it does not absorb in the visible near-infrared band (Kong et al., 2010).

Moreover, earlier studies suggested that independent scattering occurs at particle volume fractions smaller than 0.006 (Tien and Drolen, 1987). The particle volume fraction is less than 0.006 for all particle number concentrations considered in this study. The subsequent calculation results show that the microplastic suspension is a medium with strong forward scattering characteristics with an asymmetry factor above 0.92 in the visible near-infrared spectral region. Lorentz-Mie’s theory was used to theoretically calculate the SPF and asymmetry factor of the microplastics based on the complex refractive index of the materials (Peiponen et al., 2019) and the optical constants of surrounding water (Segelstein, 1981). Therefore, the absorption coefficient measurement is acceptable by using the forwardly spectral normal hemispherical transmittance, which is relatively straightforward and readily implemented under the condition that certain accuracy is guaranteed (Ma et al., 2018b; Zhao et al., 2018). Supplementary Figure S2 shows the particle size distribution for the two different microplastics with approximately spherical shapes. As shown, the average microplastic particle diameter measured was 43.41 µm and 47.78 µm for PET and PP, respectively, and the particle size distribution obtained from the measurement approximates a Gaussian distribution.

### 3.3 Experimental uncertainty and validation

The experimental uncertainty of the absorption and extinction coefficients originate from the transmittance and integration measurements. To assess the experimental uncertainty of the radiative properties of the PET, each sample was measured six times. The average value and standard deviation can be estimated by the following expression (Li et al., 2017b).

$${\bar{\xi }}_{\exp}=\frac{1}{M}\sum_{i=1}^{M}\xi_{\exp,i}$$ (7)

$$\Delta {\bar{\xi }}_{\exp}=\sqrt{\frac{1}{M-1}\sum_{i=1}^{M}{\left(\xi_{\exp,i}-{\bar{\xi }}_{\exp}\right)}^{2}}$$ (8)

respectively, where $\xi_{\exp,i}$ denotes the measured quantity, i.e., spectral transmittances and coefficients, the upper bar indicates the arithmetic mean, and *M* is the number of measurements. The experimental data were subsequently processed with error bars based on standard deviations.

The polystyrene microspheres were used to verify the accuracy of experimental measurements of the microplastic particles, which are usually used as standard particles to calibrate the experimental method. The calibration and comparison of measurement results for the experimental methods in this work have been confirmed in previous papers (Wen et al., 2023).

## 4 Results and discussions

### 4.1 Normal and hemispherical transmittance

**FIGURE 1:**
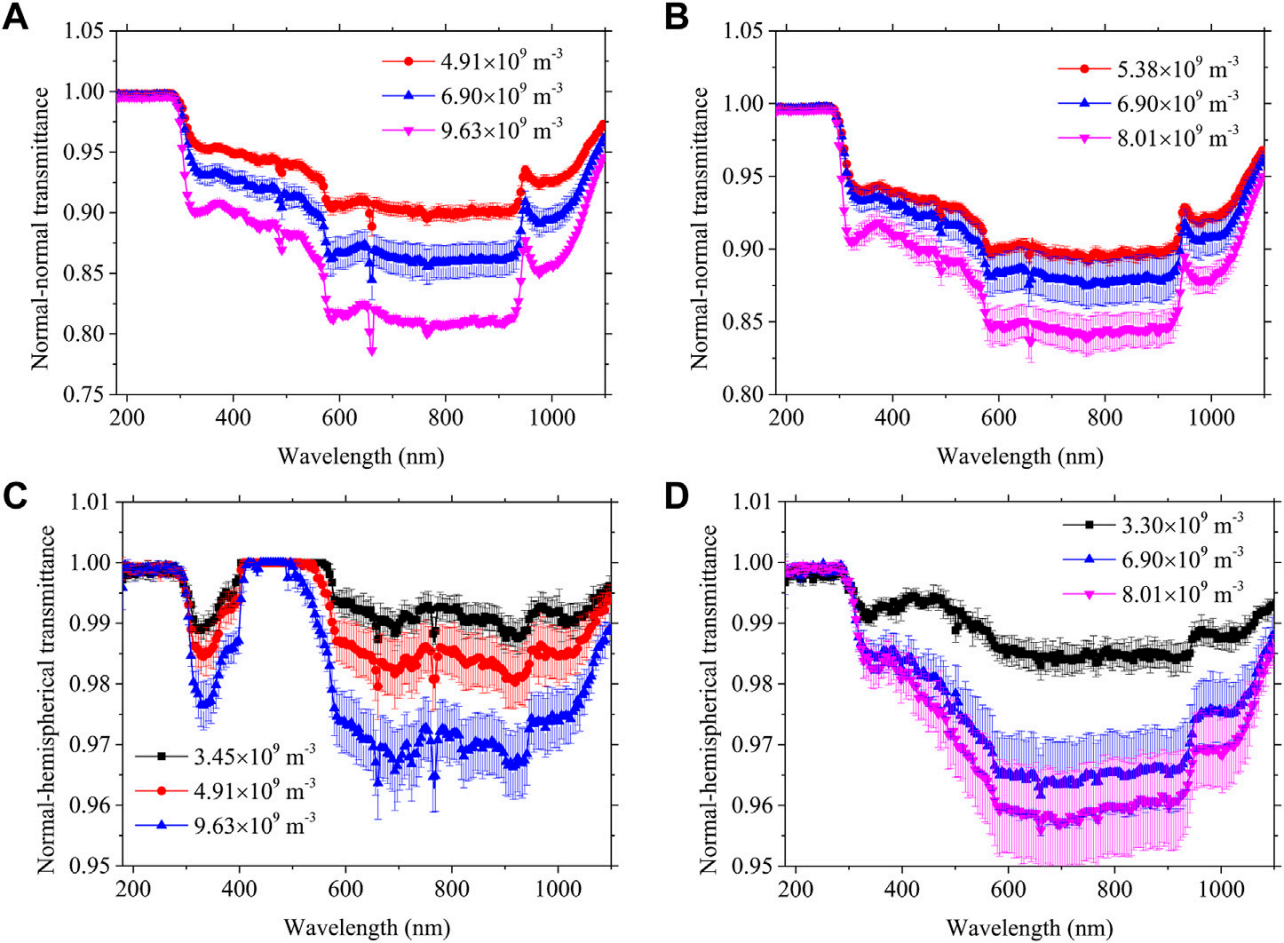
The spectral normal-normal transmittance of **(A)** PET and **(B)** PP, and the spectral normal-hemispherical transmittance of **(C)** PET and **(D)** PP.

Figure 1 shows the spectral normal-normal transmittance and normal-hemispherical transmittance at different concentrations for PET and PP, respectively. For both particles, the normal transmittance of the entire spectrum decreases with increasing particle number concentration. A low transmittance spectral band means that there is a large attenuation of light transmission. The normal transmittance trends are similar for different particle number concentrations, which indicates that the transmittance measurements meet independent scattering. Independent scattering is also the theoretical requirement that experimental measurements of absorption and extinction coefficients should satisfy. The hemispherical transmittance collects all the scattered light, so hemispherical transmittance is used to obtain absorption coefficients for translucent media or particle suspensions. The hemispheric transmittance decreases with increasing concentrations, and the transmittance at different concentrations has a similar pattern of variation. The similarity of the measurement curves for different concentrations indirectly confirms that the measurement results meet the single scattering condition.

### 4.2 Extinction coefficient and cross-section

**FIGURE 2:**
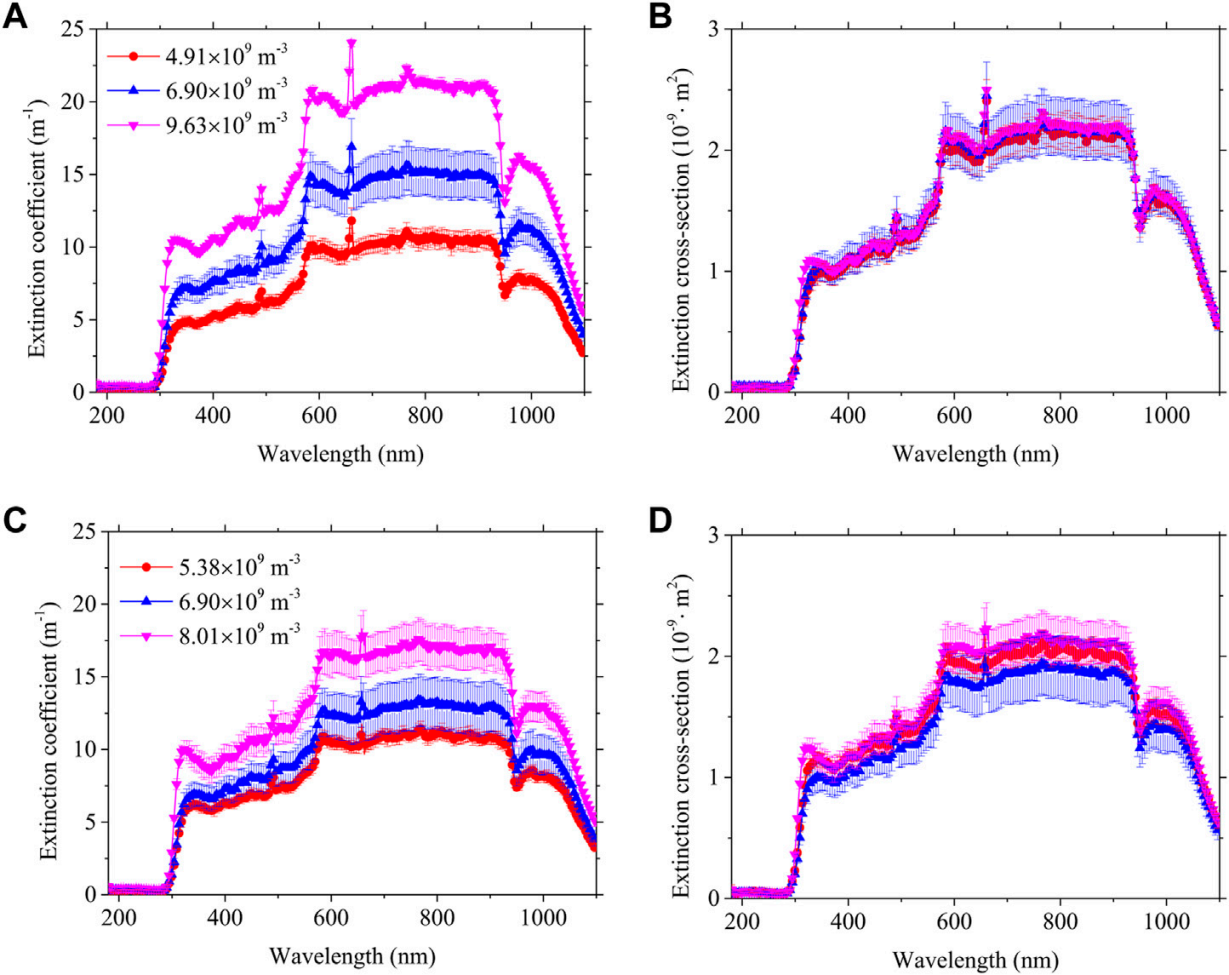
The spectral **(A)** extinction coefficient and **(B)** cross-section of PET, and the spectral **(C)** extinction coefficient and **(D)** cross-section of PP.

Figure 2 shows the measured spectral extinction coefficients and cross-sections of PET and PP in the 200–1,100 nm spectra at different concentrations with error bars. The measured extinction coefficients of PET showed similar patterns of variation at different concentrations and the extinction coefficients increased with the increase of particle number concentration. The extinction coefficient of PET reaches a maximum at 658 nm wavelength in the measured spectral range. The extinction cross-sections at different concentrations almost coincide because the extinction cross-sections are concentration independent. The optical thickness ( $\tau =\beta_{\lambda }L$ ) in the experiment satisfies the single scattering optics measurement condition of much less than one.

It also can be seen that the extinction coefficient of PP increases with increasing concentrations, while the extinction cross sections approximately overlap. The extinction cross-section is a property of the particles themselves that does not vary with concentration under independent scattering conditions. The extinction cross-sections obtained here for the three concentrations do not coincide exactly because the perfect single scattering conditions cannot be fulfilled in the actual measurement, and the experimental measurement itself is subject to errors. The extinction cross sections at different concentrations almost superimposed on each other within the experimental error.

### 4.3 Absorption coefficient and cross-section

**FIGURE 3:**
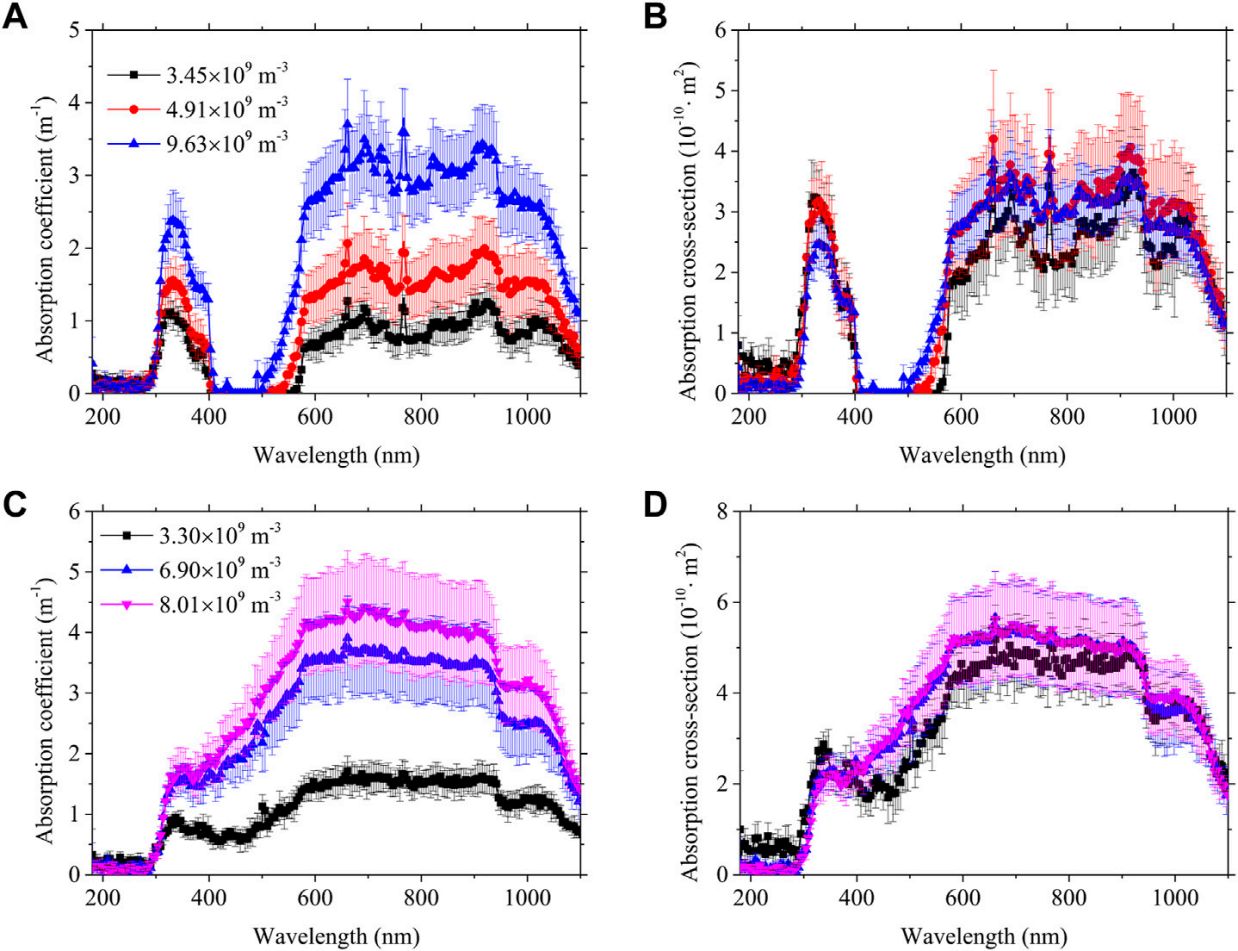
**(A)** Absorption coefficient and **(B)** absorption cross-section of PET, and **(C)** absorption coefficient and **(D)** absorption cross-section of PP.

Figure 3 shows the absorption coefficient and absorption cross-section of PET and PP obtained from the hemispherical transmittance for different particle number concentrations in the 200–1,100 nm spectrum. As shown, the absorption coefficient increases with increasing concentrations for the two microplastic particles. The absorption cross-sections of microplastic particles at different concentrations almost overlap, which is in accordance with the particle scattering theory. Similarly, the measurement of the absorption cross-section does not exactly coincide in part with the measurement error introduced by the experimental equipment and the experimental conditions. The size of the integrating sphere detection ports leads to the leakage of scattered light under the influence of the particle number concentration. The absorption peaks in the absorption coefficients at different concentrations all correspond to the same wavelength position. The absorption peaks of PET particles are more significant compared to PP particles.

The absorption cross-section of PET shows multiple significant absorption peaks near 326 nm, 700 nm, 711 nm, 767 nm, 823 nm, 913 nm, and 1,046 nm, while the absorption cross-section of PP has several significant absorption peaks in the vicinity of 334 nm, 703 nm, and 1,016 nm. Above, we only listed some obvious absorption peak locations for both microplastics; in fact, there are some absorption peaks that are not readily observable. The absorption peaks of functional groups often exist as multiple consecutive absorption peaks (Bower and Maddams, 1992). The absorption peaks of PP are less than those of PET mainly due to the fact that PP is a saturated polymer while PET is an unsaturated polymer (Jørgensen, 2015).

### 4.4 Scattering phase function

**FIGURE 4:**
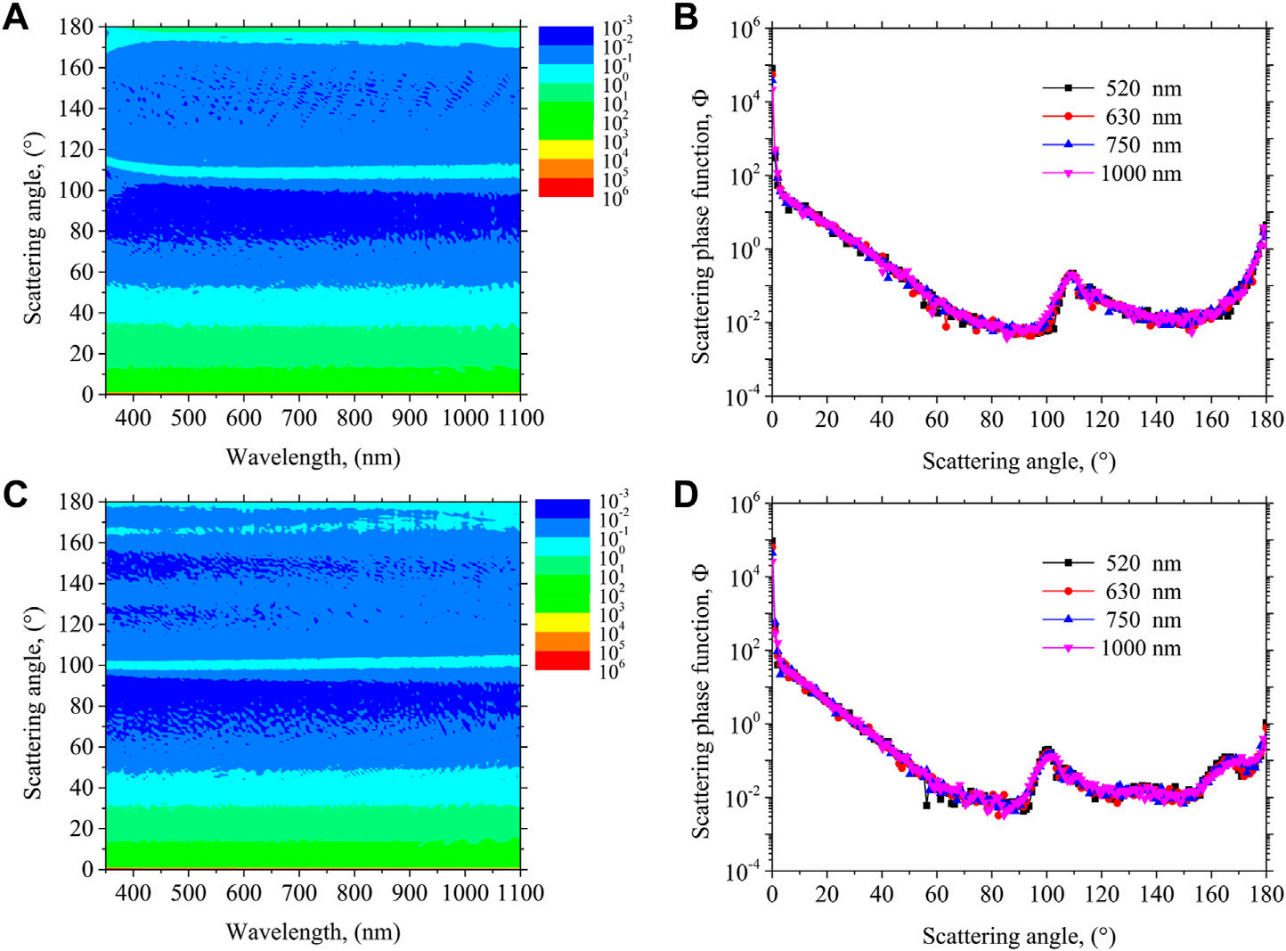
Spectral scattering phase function for PET **(A)** and PP **(C)**, and scattering phase function at four different wavelengths of PET **(B)** and PP **(D)**, respectively.

Figure 4 give the spectral SPFs and the SPFs at several typical wavelengths for PET and PP particles, respectively. The SPF of microplastic describes the spatial distribution pattern of the scattered energy. As shown, the SPFs of the two microplastic particles exhibit strong forward scattering properties, which means most scattered energy is concentrated in the forward small-angle orientation. It is also showing an enhanced backward reflection at 180° in the backward direction. The SPF of PET shows a small peak in the vicinity of 110°, and a similar peak exists for PP near 100°. The peak of PET near 110° shows a decreasing trend and then gradually increases with the increasing wavelength over the whole spectrum. Nevertheless, the peak of PP appearing near 100° shows a progressive increase with increasing wavelengths throughout the spectrum. Overall, the SPFs of PET and PP do not vary significantly over the entire Vis-NIR spectrum.

The important concept of scattering albedo should also be addressed along with the SPF. The scattering albedo is defined as the ratio between the scattering coefficient and extinction coefficient $\omega =\kappa_{s}/\beta$ , which indicates the percentage of scattered light in the light propagation process. The scattering albedo of the measured PET and PP particles above 0.7 at the entire studied spectrum indicates that the scattering of light dominates the light transfer in the Vis-NIR spectra. Thus, microplastic particles cause a significant impact on the natural light transmission processes in the water body. It is necessary for humans to pay attention to the impact of microplastics on natural ecosystems.

## 5 Conclusion

The radiative properties of PET and PP microplastics from 200 to 1,100 nm were investigated. The absorption cross-sections and extinction cross-sections obtained from measurements at different concentrations were almost coincident under independent scattering measurement conditions, respectively. The absorption cross-section of PET shows multiple significant absorption peaks near 326 nm, 700 nm, 711 nm, 767 nm, 823 nm, 913 nm, and 1,046 nm. The absorption cross-section of PP has significant absorption peaks in the vicinity of 334 nm, 703 nm, and 1,016 nm. The SPFs of the two microplastic particles exhibit strong forward scattering properties, which do not change significantly over the entire Vis-NIR spectrum. Moreover, both microplastics are scattering dominant media due to the high scattering albedo. This study provides the radiative properties of microplastics in the Vis-NIR spectrum, which can be widely employed to calculate the solar radiation distribution in a medium and create a suitable environment for microalgal photosynthesis.

## Data Availability

The raw data supporting the conclusion of this article will be made available by the authors, without undue reservation.
