# Supplementary material for: Toward a better understanding of microalgal photosynthesis in medium polluted with microplastics: a study of the radiative properties of microplastic particles
Source: Front Bioeng Biotechnol. 2023 May 4;11:1193033. doi: 10.3389/fbioe.2023.1193033 (PMC10192614; doi:10.3389/fbioe.2023.1193033)
Supplement: Supplementary file 1 [file DataSheet1.docx]

***Supplementary Material***

**Towards a better understanding of microalgal photosynthesis in medium polluted with microplastics: A study of the radiative properties of microplastic particles**

**Chunyang Ma ***

* **Correspondence**: cyma@ncu.edu.cn

Fig. S1 The three-layer optical transmission schematic of the microplastic sample for normal and hemispherical transmittance measurements.

Fig. S2 Number frequency distribution of the particle diameters of (a) PET, and (b) PP.
